# First- and Second-Line Treatments for Patients with Advanced Hepatocellular Carcinoma in China: A Systematic Review

**DOI:** 10.3390/curroncol29100575

**Published:** 2022-09-30

**Authors:** Lan Zhang, Junhui Sun, Kui Wang, Haitao Zhao, Xijie Zhang, Zhenggang Ren

**Affiliations:** 1 Liver Cancer Institute, Zhongshan Hospital, Fudan University, Shanghai 200032, China; 2 The First Affiliated Hospital, Zhejiang University School of Medicine, Hangzhou 310003, China; 3 Department of Hepatic Surgery II, Eastern Hepatobiliary Surgery Hospital, Second Military Medical University, Shanghai 200433, China; 4 Peking Union Medical College Hospital, Beijing 100032, China; 5 MRL Global Medical Affairs, MSD China, Shanghai 200233, China

**Keywords:** hepatocellular carcinoma, systemic therapy, China, systematic review

## Abstract

Chinese national guidelines recommend various systemic therapies for patients with advanced hepatocellular carcinoma (HCC), but optimal treatment selection remains uncertain. To summarize the evidence supporting the systemic treatment of Chinese patients with advanced HCC, we performed a systematic review using a literature search of PubMed, Embase, China National Knowledge Infrastructure, and the Chinese Scientific Journal Database between 1 January 2009 and 15 June 2021, and abstracts from ASCO 2020, ASCO GI 2021, ESMO 2020, and ESMO GI 2020. The inclusion criteria were: Chinese patients aged ≥18 years with advanced HCC; first- or second-line systemic therapy; an evaluation of the efficacy or safety outcomes; and a randomized controlled, non-randomized controlled, prospective, or retrospective design. Thirty reports were identified for the following therapies: the single-agent tyrosine kinase inhibitor (TKI; *n* = 10), single-agent programmed death-1 (PD-1) inhibitor (*n* = 4), chemotherapy (*n* = 5), PD-1/programmed death-ligand 1 (PD-L1) inhibitor plus TKI (*n* = 6), PD-1/PD-L1 inhibitor plus bevacizumab or biosimilar (*n* = 4), and PD-1/PD-L1 inhibitor plus chemotherapy (*n* = 1). The heterogeneity between the studies precluded statistical analysis and the data were summarized using tables. In the first-line setting, evidence supported the use of atezolizumab or sintilimab plus bevacizumab or a biosimilar. There remains insufficient evidence to determine the optimal approved TKI-based therapeutic option, and active controlled trials in the second-line setting were lacking.

## 1. Introduction

Hepatocellular carcinoma (HCC) is the sixth most common type of cancer in adults and the fourth most common cause of cancer-related mortality worldwide [1]. The pattern of HCC occurrence and mortality shows a significant geographical imbalance, predominantly due to differences in the prevalence of risk factors associated with HCC, including infection with hepatitis B virus (HBV) and hepatitis C virus (HCV) [1,2]. In China, the prevalence of HCC is particularly high, accounting for over 50% of the global HCC cases and HCC-related deaths [3]. Approximately 80% of liver cancer cases in China were attributed to chronic infection of HBV and HCV [1,4].

The therapeutic options for HCC can be divided into potentially curative (e.g., surgical tumor resection or locoregional therapy) and noncurative interventions (e.g., systemic chemotherapy). The selection of treatment is based on the stage of disease, tumor characteristics, and the presence and severity of comorbidities (e.g., liver dysfunction), among other factors [2,5]. The majority of patients with early-stage HCC are eligible for curative surgical resection, percutaneous local ablation, or liver transplantation, and those with intermediate-stage HCC can often be treated with locoregional therapies, such as transarterial chemoembolization (TACE). However, about half of patients with HCC are diagnosed with advanced-stage disease [6], equivalent to the Barcelona clinic liver cancer (BCLC) stage C or Chinese National Liver Cancer (CNLC) stage IIIa–IIIb, and characterized by vascular invasion or extrahepatic spread [7]. Patients with advanced or metastatic HCC are usually only eligible to receive systemic therapy with chemotherapy, tyrosine kinase inhibitors (TKIs), or immune checkpoint inhibitors, such as programmed death-1 (PD-1)/programmed death-ligand 1 (PD-L1) inhibitors [2,8,9]. Most international treatment guidelines recommend systemic therapy for the first- and second-line treatment of patients with advanced HCC [10]. However, differences exist between Chinese and Western patients with advanced HCC in terms of epidemiology, genetics, clinical management, and outcomes in clinical trials [11,12]. The Chinese national guidelines recommend a broader range of treatments for these patients, including locoregional therapies for selected patients [7,13]. 

Despite accumulating literature on the efficacy and safety of systemic treatments in Chinese patients with HCC, decisions about which systemic treatment should be selected as optimal based on benefit–risk considerations are not variable [13]. In this article, we analyzed the findings of a systematic collation and synthesis of the results of the studies addressing the efficacy and safety of the different systemic treatments in Chinese patients with CNLC stage IIIa–IIIb HCC. The aim of this review was to provide a summary of the current evidence supporting systemic treatments for Chinese patients with advanced HCC. This will help guide evidence-based clinical decision making on the optimal treatment of Chinese patients with advanced HCC, as well as enabling the identification of future research priorities in the field.

## 2. Materials and Methods

This systematic review is reported in accordance with the Preferred Reporting Items for Systematic Reviews and Meta-Analysis (PRISMA) 2020 guidelines [14]; the PRISMA checklist and checklist for abstracts can be found in Supplementary Files S1 and S3. The research protocol was registered at the International Prospective Register of Systematic Reviews (PROSPERO; http://www.crd.york.ac.uk/PROSPERO; protocol ID: CRD42021251536; date of registration: 5 June 2021).

### 2.1. Information Sources and Search Strategy

We searched the literature using the following databases: PubMed, Embase, China National Knowledge Infrastructure (CNKI), and the Chinese Scientific Journal Database. We also searched abstracts from ASCO 2020, ASCO GI 2021, ESMO 2020, and ESMO GI 2020. The search included English- or Chinese-language studies published from 1 January 2009 to present. A Chinese-language translated version of the search terms was used to search Chinese-language databases. The dates when each source was last searched are as follows: PubMed 20 April 2021; Embase 27 May 2021; CNKI 28 April 2021; Chinese Scientific Journal Database 15 June 2021; ESMO/ASCO June 2021.

The PubMed search strategy was “hepatocellular carcinoma” AND “intervention” (detailed in Table 1) NOT “review” NOT “preclinical” NOT “mouse” NOT “rat” AND “clinical” NOT “case” NOT “cohort” AND ““1 January 2009” [Date-Publication]: “present” [Date-Publication]”. The same search strategy was used for Embase and CNKI with the publication date settings as “2009–2021”. The search strategies for ASCO and ESMO were simplified because of the scope of these databases and involved searching “hepatocellular carcinoma” AND “intervention” (detailed in Table 1). The Chinese Scientific Journal Database search was conducted in Chinese with the translations of the search terms shown in Table 2 and Table 3. The detailed search strategies are listed in Supplementary File S2.

### 2.2. Study Selection

Records collected during the search were independently assessed for inclusion by two reviewers against predefined inclusion and exclusion criteria. Disagreements were resolved by discussion. The inclusion criteria were: (1) Chinese patients aged ≥18 years with HCC CNCLC IIIa–IIIb or BCLC stage C; (2) first- or second-line treatment with the interventions described in Table 1; (3) study/treatment of ≥1 year duration; (4) evaluation of ≥1 of the following outcomes: overall response rate (ORR), progression-free survival (PFS), overall survival (OS), duration of response (DOR), or safety; (5) randomized controlled trials (RCTs), non-randomized controlled trials, prospective or retrospective comparative studies, and single-arm studies (if comparative studies were not available). Studies were excluded if they met any of the following criteria: (1) reviews, case series, case reports, editorials, and letters; (2) data on Chinese patients could not be extracted from pooled results; (3) patients with HCC not classified as CNCLC stage IIIa–IIIb or BCLC stage C.

### 2.3. Data Collection and Data Items

Full-text articles were obtained for records that met the inclusion criteria, and two reviewers independently extracted data from the full-text articles of the included studies. Data from the relevant publications were extracted using standardized data extraction tables. We extracted the following data from included articles: (1) author names, year of publication, country of publication, and geographical setting; (2) study design; (3) study size (number of centers and patients/participants); (4) patient demographics and characteristics (including age, sex, HCC stage); (5) interventions (treatment, dosage, and duration); (6) outcomes and follow-up time points; and (7) data for quality and risk of bias assessment. Disagreements were resolved by discussion and with assistance from a third party (Jake Burrell), if required, until a consensus was formed. Data from studies reported in Chinese language were extracted by two native Chinese speakers.

### 2.4. Assessment of the Risk of Bias and Certainty in Individual Studies

Characteristics of the studies used to assess bias included random sequence generation (risk of selection bias), allocation concealment (risk of selection bias), incomplete outcome data (risk of attrition bias), selective outcome reporting, blinding of participants and personnel (performance bias and detection bias), and heterogeneity in baseline characteristics and outcome measurements. We evaluated risk of bias at the study level using the Cochrane Collaboration’s tools for assessing the risk of bias [15,16]. Two reviewers independently assessed the risk of bias in each individual study. Disagreements were resolved by discussion, with support from a third party (Jake Burrell) if required.

Risk of bias for RCTs was evaluated using the RoB2 tool, which included five domains: (1) method of randomization, (2) deviations from intended interventions involving (a) the effect of assignment to the invention and (b) the effect of adhering to the intervention, (3) missing outcome data, (4) measurement of the outcome, and (5) selection of the reported result. For each domain, the studies were ranked as low, some concerns, or high. For non-randomized trials with ≥2 arms, the ROBINS-I tool was used, which evaluates the risk of bias based on seven factors: (1) confounding, (2) selection of participants, (3) classification of interventions, (4) intended interventions, (5) missing data, (6) measurement of outcomes, and (7) selection of reported result. For each factor, the studies were classified as low, moderate, serious, or critical. Single-arm trials were not formally evaluated for bias, as they do not compare outcomes.

### 2.5. Measurements of Effect

We organized results from trials by intervention type and treatment effect/outcome. The main outcomes were: (1) ORR (proportion of patients with a complete or partial response by imaging assessment using the response evaluation criteria in solid tumors (RECIST) or modified RECIST (mRECIST) criteria); (2) PFS (time from randomization or assignment to treatment to disease progression or death from any cause); (3) DOR (time from disease response to disease progression); (4) OS (time from randomization or inclusion to death from any cause); (5) severe (grade ≥ 3) adverse events per the National Cancer Institute Common Terminology Criteria for Adverse Events. ORR data were summarized as proportion of patients and 95% confidence intervals (CIs). PFS, OS, and DOR were summarized as medians and 95% CIs. Safety data were summarized as number and percentage of patients with treatment-related adverse events (TRAEs) or treatment-emergent adverse events (TEAEs). 

### 2.6. Data Synthesis

Due to the significant variation in patient characteristics, clinical settings, interventions, and reported outcomes among the included studies, we did not quantitively combine data in a meta-analysis. Instead, we conducted a qualitative synthesis by summarizing the extracted data in tables. Along with the narrative report of the outcome data of each study, we also summarized the quality and potential for bias of each data source.

## 3. Results

### 3.1. Study Selection

**Figure 1:**
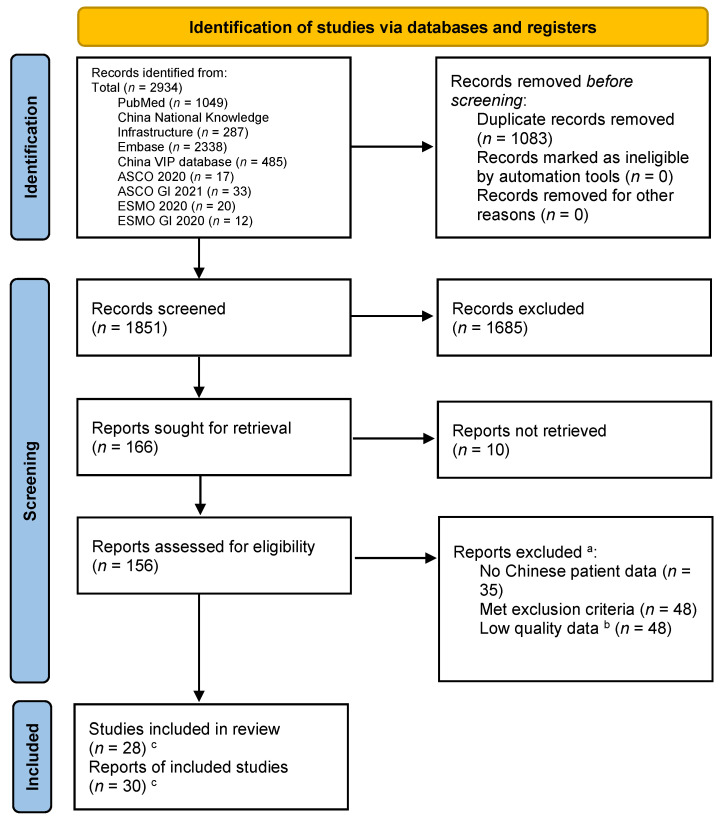
PRISMA flow diagram of study identification and selection. ^a^ Studies could have been excluded for more than one reason. ^b^ The Chinese language searches returned a large volume of studies with small numbers of patients and with designs likely to have introduced a high level of bias and imprecision. For each treatment with data available, only the highest quality studies were selected for inclusion in this review. ^c^ Five studies were added during the writing of the review, because from the title and abstract, they did not appear to meet the inclusion criteria but on inspection of the full text were suitable for inclusion or were published after the initial literature search was completed. Two studies had multiple reports.

The searches of the PubMed, Embase, CNKI, VIP, ASCO 2020, ASCO GI 2021, ESMO 2020, and ESMO GI 2020 databases returned a total of 2934 records (Figure 1). Of these, 1083 records were duplicates and were removed before screening. Of the remaining 1851 records screened, 1685 records were discarded after assessing the titles and abstracts for inclusion (Figure 1). The full-text articles of the remaining 166 citations were sought for retrieval for a detailed evaluation, and 156 full-text reports (including congress abstracts) could be retrieved. Of these, 35 lacked extractable Chinese patient subgroup data, 48 met the exclusion criteria upon detailed evaluation, and 48 were excluded due to a serious risk of bias. Finally, 30 reports were included in the systematic review, including 5 that were added during the writing of this review (Table 4). No further studies were identified by screening the references of the included articles.

### 3.2. Study Characteristics

The key characteristics of the 30 reports included in this systematic review are shown in Table 4, and the details of the interventions used are summarized in Table 5. Overall, 28 studies were included as 2 studies were reported twice in two separate publications. Overall, 36% (10/28) of the studies were randomized, 50% (14/28) included an active control arm, and 2 were placebo controlled, while 64% (18/28) were non-randomized, 54% (15/28) were single arm, 43% (12/28) were single center, and 64% (18/28) included a sample of <100 patients. The mean/median patient ages were between 46 and 60 years, and the proportion of male patients ranged between 74.1 and 100%.

### 3.3. Effects and Safety of Interventions

The treatment outcomes and safety for all included studies are summarized in Table 6.

#### 3.3.1. TKI Monotherapy

Based on the global multicenter studies that included Chinese patients, sorafenib, lenvatinib, and regorafenib have been approved as first- and second-line treatments for advanced stage HCC. In this review, nine studies of TKI monotherapy in Chinese patients were identified, including one study with two separate reports [17,18,19,20,21,22,23,24,25,26]. Of these, 33% (3/9) were randomized, 56% (5/9) were single arm, and 89% (8/9) had a prospective design. Across all studies, where reported, the ORR ranged from 4.6 to 40.9% (by RECIST or RECIST 1.1), the median PFS ranged from 3.0 to 6.8 months, and the median OS ranged from 5.0 to 12.1 months. When considered by individual TKIs, the ORR and OS, respectively, where available, were 10.7–40.9% and 8.7 months with apatinib [17,22,25], 22% and not reported with lenvatinib [19], 7.8% and 5 to 11.3 months with sorafenib [20,21,26], 9.1% and 5.36 months with cabozantinib [23], and 4.6–4.8% and 12.1 months with donafenib [18,24].

There was one head-to-head comparison of different TKIs [18]. In this study, donafenib demonstrated a superior median OS compared with sorafenib as a first-line treatment for patients with unresectable or metastatic HCC (12.1 vs. 10.3 months; HR 0.831; 95% CI 0.699, 0.988; *p* = 0.0245) [18]. However, the ORR (4.6 vs. 2.7%; *p* = 0.2488) and median PFS (3.7 vs. 3.6 months; HR 0.909; 95% CI 0.763, 1.082; *p* = 0.0570) were similar between the two arms. The incidence of grade ≥ 3 TRAEs was significantly lower with donafenib compared with sorafenib (38 vs. 50%; *p* = 0.0018). 

In a randomized, placebo-controlled study, apatinib significantly prolonged the PFS (4.5 vs. 1.9 months; HR 0.471; 95% CI 0.369–0.601; *p* < 0.0001) and OS (8.7 vs. 6.8 months; HR 0.785; 95% CI 0.617–0.998; *p* = 0.048) compared with the placebo in patients who had received at least one line of systemic therapy [22]. The incidence of grade 3 or 4 TRAEs was 77% with apatinib versus 19% with placebo [22].

#### 3.3.2. PD-1/PD-L1 Inhibitor Monotherapy 

Four articles were identified reporting outcomes of immune checkpoint inhibitor monotherapy, of which two reported results from a multicenter phase II trial of two dosing regimens of camrelizumab in patients who had previously received systemic treatment [27,28]. In the total population, the ORR was 14.7% and the median OS was 13.8 months [27]. The incidence of grade 3 or 4 TRAEs was 22%. A longer-term follow-up analysis of this study demonstrated a median OS of 14.2 months [28].

Two articles were recent congress abstracts. The first abstract reported findings from a phase III study of pembrolizumab as a second-line therapy (KEYNOTE-394) [29]. Pembrolizumab compared with placebo improved the median OS (14.6 vs. 13.0 months; HR 0.79; 95% CI 0.63–0.99; *p* = 0.0180), median PFS (2.6 vs. 2.3 months; HR 0.74; 95% CI 0.60–0.92; *p* = 0.0032), and ORR (13.7 versus 1.3%). Pembrolizumab compared with placebo had a higher incidence of any TRAEs (66.9 vs. 49.7%) and grade ≥ 3 TRAEs (14.4 vs. 5.9%). The second abstract reported findings from a phase II, open-label study of tislelizumab as a second- or third-line treatment [30]. Tislelizumab was associated with a median OS of 13.5 months, median PFS of 2.7 months, and ORR of 13.6%. The incidences of any and grade ≥ 3 TRAEs were 62.6 and 13.6%, respectively.

#### 3.3.3. Chemotherapy

Of the five identified studies of chemotherapy alone [31,32,33,34,35], two randomized patients to two different chemotherapy regimens, two had a single-arm design, and one compared different doses of pegylated arginine deiminase (ADI-PEG 20). All studies reported an ORR, which ranged from 0% with ADI-PEG 20 to 20% with FOLFOX4. The median PFS ranged from 1.8 to 2.9 months, and the median OS ranged from 5.0 to 9.7 months. In the four studies investigating oxaliplatin-based chemotherapies, the ORRs ranged from 8.2 to 20.0%, suggesting a similar efficacy among these various regimens [32,33,34,35]. In one study, there was a trend toward an increased OS with FOLFOX4 compared with doxorubin (6.4 vs. 5.0 months; HR 0.80; 95% CI 0.63–1.02; *p* = 0.07), as well as a significant improvement in PFS (2.9 vs. 1.8 months; HR 0.62; 95% CI 0.49–0.79; *p* < 0.001) and ORR (8.2 vs. 2.7%; *p* = 0.02) [35]. 

#### 3.3.4. PD-1/PD-L1 Inhibitor plus Tyrosine Kinase Inhibitor 

No randomized prospective controlled studies investigating the efficacy and safety of PD-1/PD-L1 in combination with a TKI were identified. However, six studies investigating the combination of a PD-1/PD-L1 inhibitor plus a TKI were retrieved, including four single-arm prospective studies [36,37,38,39] and two retrospective studies [40,41]. It should be noted that the study sample sizes were small and further robust studies with larger patient populations are warranted. Across all studies, the ORR ranged from 21.4% (by mRECIST) to 42.9% (by RECIST). A median PFS was reported from four studies and ranged from 5.5 to 8.8 months [37,39,40,41]. In a study of sintilimab plus anlotinib, the PFS rate at 6 months was 78.8% and the median PFS was not reached [36]. The available data suggest that the responses were durable. OS was reported from two studies of camrelizumab plus apatinib [37,41]. In the first, a multicenter, prospective study, the 18-month OS rates were 58.1 and 56.5% with first- and second-line treatment, respectively [37]. The second, a multicenter, retrospective study, reported a 12-month OS rate of 62.3% with second-line treatment [41]. In the only study with a comparator arm, camrelizumab plus sorafenib improved the ORR (by mRECIST, 24.0 vs. 4.0%; *p* = 0.025) and median PFS (8.0 vs. 6.4 months; *p* = 0.040) compared with sorafenib alone, while the median OS was similar between the two groups (7.4 vs. 7.0 months; *p* = 0.513) [40]. Across the four studies with available data, the incidences of grade ≥ 3 TRAEs varied considerably, ranging from 19.4% with penpulimab plus anlotinib [39] and 77.4% with camrelizumab plus apatinib [37]. This variation probably reflects the small study sample sizes.

#### 3.3.5. PD-1/PD-L1 Inhibitor plus Bevacizumab or Biosimilar

The efficacy and safety data were recently reported from the Chinese subpopulation of the randomized, phase III IMbrave150 trial of atezolizumab plus bevacizumab versus sorafenib in patients with systemic treatment-naïve unresectable HCC [42]. In this analysis, atezolizumab plus bevacizumab was associated with clinically meaningful improvements compared with sorafenib in terms of the OS (median, not reached versus 11.4 months; stratified HR 0.44; 95% CI 0.25–0.76) and PFS (median, 5.7 versus 3.2 months; stratified HR 0.60; 95% CI 0.40–0.90). The ORR was 24.6 versus 6.7% (by RECIST 1.1), respectively. Any grade TRAEs (90.2 vs. 93.1%) and grade 3 or 4 TRAEs (43.9 vs. 37.9%) occurred at a similar frequency in the atezolizumab plus bevacizumab versus sorafenib groups, respectively.

In addition, three studies assessed sintilimab plus a bevacizumab biosimilar (IBI305) in the first-line setting [43,44,45]. In a large, randomized phase II/III study, sintilimab plus IBI305 compared with sorafenib monotherapy was associated with a higher ORR (21 versus 4%; by RECIST 1.1, *p* < 0.0001) and longer median PFS (4.6 versus 2.8 months; HR 0.56; 95% CI 0.46–0.70; *p* < 0.0001) and median OS (not reached versus 10.4 months; HR 0.57; 95% CI 0.43–0.75; *p* < 0.0001) [43]. The incidence of grade ≥3 TEAEs was similar in the sintilimab plus IBI305 and sorafenib groups (54.0 vs. 47.0%). These findings were supported by preliminary data from the two smaller phase I/II studies [44,45].

#### 3.3.6. PD-1/PD-L1 Inhibitor plus Chemotherapy

A single-arm phase Ib/II study of camrelizumab plus FOLFOX4 as a first-line systemic therapy in advanced HCC patients was identified [46]. The ORR was 29.4%, and the median DOR was 6.9 months (range, 3.3–11.5). The PFS and OS were 7.4 months (95% CI, 3.9–9.2) and 11.7 months (95% CI, 8.2–22.0), respectively. Grade ≥ 3 TRAEs were reported at an incidence of 85.3%.

#### 3.3.7. Chemotherapy plus Targeted Agents

There were no published articles on chemotherapy plus targeted agents that met the inclusion criteria, highlighting the lack of high-quality evidence for this treatment modality in advanced HCC.

## 4. Discussion

This systematic review provides a summary of the available evidence concerning the efficacy and safety of different systemic treatments in Chinese patients with advanced unresectable HCC. Of the various systemic treatments, the largest number of studies were retrieved for TKIs administered as monotherapy, but most lacked a comparator arm. One active-controlled study suggested that donafenib may offer superior OS outcomes and a lower incidence of grade ≥3 TRAEs compared with sorafenib [18]. A placebo-controlled study also indicated survival benefits with apatinib in the second-line setting [22]. It should be noted that this was the only placebo-controlled study identified in our search. Although placebo arms are generally not included in cancer treatment trials for ethical reasons, there was no approved standard of care in the second-line setting at the time this study was being conducted. The findings from global phase III trials that included Asian patients but did not meet the eligibility criteria for this review also support the use of TKIs in Chinese patients with advanced HCC [8,9,47,48].

We found three studies assessing immune checkpoint inhibitors as monotherapy, which suggested encouraging efficacy with camrelizumab, pembrolizumab, or tislelizumab as the second-line treatment [27,28,29,30]. The international phase I/II CheckMate 040 study, which included centers in Asia, demonstrated the efficacy of nivolumab monotherapy as a second-line treatment in patients with advanced HCC [49]. However, the phase III CheckMate 459 study showed that nivolumab did not significantly improve overall survival compared with sorafenib in the first-line setting [50]. The phase II KEYNOTE-224 study, which did not include Chinese patients, initially showed efficacy of pembrolizumab monotherapy in the second-line setting [51]. However, in the phase III KEYNOTE-240 study, which compared second-line pembrolizumab monotherapy with placebo in patients with advanced HCC, results from the primary endpoints (OS and PFS) did not reach the prespecified criteria for statistical significance, although the benefit-to-risk ratio for pembrolizumab was favorable [52]. A post hoc analysis of KEYNOTE-240 showed a trend toward a greater efficacy benefit in the Asian subpopulation versus the overall cohort [53], consistent with the significant improvements in the OS, PFS, and ORR observed with pembrolizumab versus placebo in the KEYNOTE-394 study in Asian patients [29]. 

Several studies on single chemotherapy regimens were retrieved, which supported the use of oxaliplatin-based chemotherapies in advanced HCC, with no clear advantages in favor of a particular regimen. Among the studies investigating combinations of different treatment types, the strongest evidence was provided by the sub-analysis of the phase III IMbrave150 study, which demonstrated improved efficacy outcomes with atezolizumab plus bevacizumab compared with sorafenib in Chinese patients [42]. Moreover, a large, randomized phase II/III study (ORIENT 32) showed improved efficacy outcomes with a similar type of treatment combination, sintilimab plus IBI305, over sorafenib in the first-line setting [43]. In addition, promising clinical activity with PD-1/PD-L1 inhibitors combined with TKIs in the first- or second-line setting was suggested in single-arm studies [36,37,38,39,40,41]. However, these findings need to be confirmed in randomized studies with larger sample sizes. A small study also suggested the potential antitumor activity of camrelizumab combined with FOLFOX4 for the first-line treatment of advanced HCC [46]. 

Other than the results from the Chinese sub-analysis of the phase III IMbrave 150 trial [42] and the phase II/III ORIENT 32 trial [43], no randomized controlled evidence was available for combination strategies, such as PD-1 antibodies in combination with a TKI or chemotherapy in Chinese patients. Evidence from single-arm, studies with a small sample size have limited strength, and further phase III randomized studies are warranted for robust evidence. In this review, a data synthesis was precluded due to the clinical heterogeneity detected between studies during the feasibility assessment, and therefore it is not possible to draw any definitive conclusions regarding the relative efficacy of different treatment types from this qualitative review. However, the compiled efficacy and safety information can be used as a reference for clinical practice. In general, the available data in Chinese patients with advanced HCC support the use of combination therapy with a PD-1/PD-L1 inhibitor (atezolizumab or sintilimab) and bevacizumab in the first-line setting compared with sorafenib.

Although the BCLC staging system is widely preferred for staging HCC, the restrictive criteria for treatment recommendation and allocation have been challenged [54,55]. Unlike the BCLC staging system, which categorizes patients with advanced HCC into one category (stage C), the CNLC system divides these patients into two subclasses (stages IIIa and IIIb) [7]. Reflecting these differences in disease classification, the range of recommended treatments for advanced HCC is more restrictive in international guidelines compared with Chinese national guidelines. The Chinese guidelines provide a variety of treatment options based on the disease stage and individual characteristics of patients [7,13]. 

According to international guidelines, systemic therapy with atezolizumab plus bevacizumab is the preferred first-line treatment for patients with advanced HCC and Child–Pugh class A liver function [10,56,57]. This recommendation was based on results from the phase III IMbrave150 trial, which demonstrated a significant OS benefit for atezolizumab plus bevacizumab compared with sorafenib in this setting (median OS, 19.2 vs. 13.4 months; HR, 0.66; 95% CI 0.52–0.85; descriptive *p* < 0.001) [58]. The median PFS was also significantly prolonged with atezolizumab plus bevacizumab (6.9 vs. 4.3 months; HR 0.65; 95% CI 0.53–0.81; descriptive *p* < 0.001), and the ORR (RECIST v1.1) was 27.3 versus 11.9%, respectively (*p* < 0.001) [59]. If there are contraindications to atezolizumab plus bevacizumab, the guidelines state that sorafenib or lenvatinib may be offered as an alternative first-line treatment [10,56,57]. For patients with disease progression on first-line therapy, recommended second-line options usually involve TKI therapy with sorafenib, lenvatinib, regorafenib, ramucirumab, or cabozantinib, while immune checkpoint inhibitors may be considered for patients with progression on or intolerance to TKIs.

In contrast, Chinese guidelines endorse a wider range of treatments for patients with advanced HCC, including systemic therapy with TKIs, FOLFOX4, or PD-1 inhibitors; TACE for CNLC stage IIIa and select IIIb cases; and resection with or without radiotherapy for CNLC stage IIIa cases [7,13]. TACE in combination with TKIs or immunotherapy is also recommended [7]. The recommended first-line systemic treatments in China consist of sorafenib, lenvatinib, or oxaliplatin-based chemotherapy, while regorafenib or PD-1 inhibitors are recommended in the second-line setting [13]. In addition, donafenib and apatinib, which were independently developed in China, have recently been approved by the China National Medical Products Administration (NMPA) as first- and second-line treatments for advanced HCC. Combined immuno-oncology options, including a PD-1 inhibitor (sintilimab) and a PD-L1 inhibitor (atezolizumab) in combination with bevacizumab or biosimilar, have been approved as first-line options by the NMPA. 

A key limitation of this review was the considerable proportion of the included studies that were published only as conference abstracts, of which the data collected had therefore not undergone peer review and the risk of reporting bias was increased due to missing results. Another limitation was that some studies did not specify whether the systemic therapy under investigation was given in the first- or second-line setting or included patients regardless of therapy line. 

Clinical decisions for the treatment of HCC are complex, integrating the tumor burden, disease stage and aggressiveness, and patient characteristics, such as age, existing comorbidities, and liver dysfunction. This is particularly true for the treatment of advanced HCC using systemic interventions, which can aggravate underlying liver conditions. Variability in the available treatment options and level of expertise and resources further complicates the management of patients with advanced HCC [2]. There is a clear need for further head-to-head studies in Chinese patients to guide clinical decisions given the range of available systemic treatment choices, as well as evidence regarding the optimal sequencing of therapies.

## 5. Conclusions

The available evidence in Chinese patients with advanced HCC supports the first-line use of atezolizumab or sintilimab plus bevacizumab or a biosimilar, as these regimens have shown superior efficacy versus sorafenib in this patient population. However, TKIs and oxaliplatin-based chemotherapy have demonstrated survival benefits and remain as options for first-line treatment, depending on individual patient characteristics. There is currently insufficient evidence to determine a preferred second-line systemic treatment, which should be selected according to individual patient situations. Although the heterogeneity of the data precluded conducting a meta-analysis, this review provides a summary of the landscape of the available evidence for systemic treatment in Chinese patients with advanced HCC, which will support clinical decision making and inform future research. Further head-to-head controlled trials between different regimens in different populations, including first-line, and TKI- or immuno-oncology- exposed second-line patients, are encouraged.

## Figures and Tables

**Table 1:** Search terms for Chinese National Knowledge Infrastructure.

Search Terms
Hepatocellular
AND intervention
NOT review
NOT preclinical
NOT mouse
NOT rat
NOT Transarterial
NOT TACE
NOT surgery
Date range: 2008–present

**Table 2:** Interventions for Chinese National Knowledge Infrastructure search.

Intervention
**Tyrosine kinase inhibitors**
Lenvatinib
Sorafenib
Cabozantinib
Erlotinib hydrochloride
Tepotinib/MSC2156119J
Regorafenib
Tivantinib/ARQ 197
Apatinib
Donafenib
Anlotinib
Milciclib maleate
**Anti-angiogenic therapy**
Bevacizumab
Ramucirumab
**PD-1/PD-L1 inhibitors**
Nivolumab
Atezolizumab
Pembrolizumab
Durvalumab
Camrelizumab/SHR-1210
Sintilimab
Tislelizumab
Toripalimab
**Other immune checkpoint inhibitors**
Ipilimumab
Tremelimumab
**Chemotherapy**
mFOLFOX/FOLFOX
Doxorubicin
CF-102/namodenoson
Capecitabine
Lipotecan/TLC388
Yeliva/opaganib
Pegylated arginine deiminase/ADI-PEG20
Mipsagargin

**Table 3:** Interventions included in literature searches.

Tyrosine Kinase Inhibitors	Anti-Angiogenic Agents	PD-1/PD-L1 Inhibitors	Other Immune Checkpoint Inhibitors	Chemotherapy
Lenvatinib	Bevacizumab	Nivolumab	Ipilimumab	mFOLFOX/FOLFOX
Sorafenib	Ramucirumab	Atezolizumab	Tremelimumab	Doxorubicin
Cabozantinib		Pembrolizumab		CF-102/namodenoson
Erlotinib hydrochloride		Durvalumab		Capecitabine
Tepotinib/MSC2156119J		SHR-1210/camrelizumab		Lipotecan/TLC388 ^a^
Regorafenib		Sintilimab		Yeliva/opaganib
Tivantinib/ARQ 197		Tislelizumab		Pegylated arginine deiminase/ADI-PEG20
Apatinib		Toripalimab		Mipsagargin
Donafenib				
Anlotinib				

^a^ No results found.

**Table 4:** Characteristics of the included studies.

Study	Period	Study Design	No. of Centers	No. of Patients	BCLC Distribution (A:B:C:D), %		Age, Years		Sex, Male, *n* (%)		Treatment Line	Intervention	Comparator
					I	C	I	C	I	C			
**Tyrosine Kinase Inhibitor**													
Zhang et al. [17]	Apr 2017–Jul 2018	Non-randomized	1	16	-	-	-	-	-	-	1 L	Apatinib	-
Qin et al. [18]	2016–2019	Randomized	37	665	0:13:87:0	0:12:88:0	53 (46–62) ^a^	53 (46–61) ^a^	281 (86)	291 (88)	1 L	Donafenib	Sorafenib
Wang et al. [19]	Dec 2018–Dec 2019	Non-randomized	1	54	0:33.3:66.7:0	-	58.94 ± 12.10 ^b^	-	46 (85.2)	-	NS	Lenvatinib	-
Ye et al. [20]	2008–2016	Non-randomized	75	338	22.8:67.5 ^c^	-	50 ± 11.5 ^b^	-	312 (92.3)	-	NS	Sorafenib	-
Ye et al. [21]	2008–2016	Non-randomized	75	338	5.3:17.5:62.1:5.3	-	50 ^d^	-	312 (92.3)	-	NS	Sorafenib	-
Qin et al. [22]	2014–2017	Randomized	31	400	0:11:89:0	0:8:92:0	51 (27–78)	50 (25–77)	223 (85)	116 (88)	≥2 L	Apatinib	Placebo
Dong et al. [23]	-	Non-randomized	^e^	22	-	-	57.1 (48.5–58.6 ^f^	-	-	-	>70% 2 L	Cabozantinib	Cabozantinib + ICI
Bi et al. [24]	2014–2015	Randomized	10	106	-	-	-	-	-	-	1 L	Donafenib	Donafenib
Kong et al. [25]	Sep 2015–Oct 2016	Non-randomized	1	22	0:0:100:0	-	54.3 (32–77) ^f^	-	19 (86.4)	-	1 L or 2 L	Apatinib	-
Yau et al. [26]	2006–2008	Non-randomized	1	51	-	-	56 (28–79) ^f^	-	45 (88)	-	1 L or 2 L	Sorafenib	-
**PD-1/PD-L1 Inhibitor**													
Qin et al. [27]	2016–2017	Randomized	13	217	0:5:95:0	-	49 (41–59) ^a^	-	196 (90)	-	≥2 L	Camrelizumab	-
Ren et al. [28]	2016–2017	Randomized	-	172	-	-	-	-	-	-	≥2 L	Camrelizumab	-
Qin et al. [29]	Apr 2017–Jun 2021	Randomized	41	453	92.3 (C stage)	95.4 (C stage)	54 (22–82)	54 (22–78)	257 (85.7)	126 (82.4)	2 L	Pembrolizumab	Placebo
Edeline et al. [30]	Apr 2019–Feb 2022	Non-randomized	65	249	-	-	-	-	-	-	≥2 L	Tislelizumab	-
**Chemotherapy**													
Yang et al. [31]	2006–2008	Randomized	8	71	-		55 (27–82) ^f^	-	59 (83.1)	-	1 L or ≥2 L	ADI-PEG20	-
Zhu et al. [32]	2009–2011	Non-randomized	1	20	-	-	52.5 (45–60) ^h^	-	20 (100)	-	2 L	FOLFOX4	-
Jiang et al. [33]	Feb 2013–Apr 2017	Randomized	1	53	-	-	56 (32–71) ^f^	55 (33–69) ^f^	20 (74.1)	19 (73.1)	NS	XELOX	FOLFOX
Zhang et al. [34]	Jan 2005–Dec 2006	Non-randomized	1	31	-	-	46 (38–58) ^f^	-	29 (93.6)	-	NS	FOLFOX6	-
Qin et al. [35]	Mar 2007–May 2009	Randomized	38	371	0:21.2:78.8:0	0:18.7:81.3:0	49.53 ± 10.77	49.30 ± 10.80	166 (90.2)	163 (87.2)	1 L or >1 L	FOLFOX4	Doxorubicin
**PD-1/PD-L1 Inhibitor + Tyrosine Kinase Inhibitor**													
Chen et al. [36]	Ongoing	Non-randomized	1	16	0:18.8:81.3:0	-	56 (41–69) ^f^	-	14 (87.5)	-	1 L	Sintilimab + anlotinib	-
Xu et al. [37]	2018–2019	Non-randomized	25	70 (first-line), 120 (second-line)	0:17.1:82.9:0	0:18.3:81.7:0	53 (44–60) ^a^	51 (43–58) ^a^	63 (90.0)	106 (88.3)	1 L or 2 L	Camrelizumab + apatinib	-
Liu et al. [38]	Jan 2019–Dec 2019	Non-randomized	1	90	0:60.0:40.0:0	0:68.0:320:0	51.9 ± 9.0 ^g^	53.5 ± 6.6 ^g^	20 (80.0)	38 (76.0)	NS	Camrelizumab + sorafenib	Sorafenib
Lin et al. [39]	Ongoing	Non-randomized	^e^	30 (14 evaluable)	-	-	-	-	-	-	1 L	Anlotinib + toripalimab	-
Han et al. [40]	Ongoing	Non-randomized	8	31	0:23:77:0	-	56 (23–74) ^e^	-	25 (80.6)	-	1 L	Penpulimab + anlotinib	-
Yuan et al. [41]	Feb 2019–Feb 2020	Non-randomized	5	94	0:16.0:84.0:0	-	52.7 ± 12.3 ^g^	-	87 (92.6)	-	2 L	Camrelizumab + apatinib	-
**PD-1/PD-L1 Inhibitor + Bevacizumab or Biosimilar**													
Qin et al. [42]	Apr 2018–Apr 2019	Randomized	28	194	2.3:11.3:86.5:0	1.6:4.9:93.4:0	57 (29–82) ^f^	60 (31–82) ^f^	116 (87.2)	49 (80.3)	1 L	Atezolizumab + bevacizumab	Sorafenib
Ren et al. [43]	2019–2020	Randomized	50	571	0:15:85:0	0:14:86:0	53 (21–82) ^f^	54 (28–77) ^f^	334 (88)	171 (90)	1 L	Sintilmab + IBI305	Sorafenib
Jia et al. [44]	Ongoing	Non-randomized	1	24	-	-	-	-	-	-	1 L	Sintilimab + IBI305	-
Zhang et al. [45]	Ongoing	Non-randomized	1	50	-	-	-	-	-	-	1 L	Sintilimab + IBI305	Sintilimab + IBI305
**Chemotherapy + PD-1/PD-L1 Inhibitor**													
Li et al. [46]	Ongoing	Non-randomized	6	34	0:4:29:0	-	52 (36–70)	-	31 (91.2%)	-	1 L	Camrelizumab + FOLFOX4	-

^a^ Median (interquartile range). ^b^ Mean (standard deviation). ^c^ BCLC A and B: C and D. ^d^ Median. ^e^ Multicenter study, but the number of centers is not reported. ^f^ Median (range). ^g^ Units not stated. ^h^ Mean (range), 5-FU: 5-fluorouracil; ADI-PEG 20: Arginine deiminase-polyethylene glycol; BCLC: Barcelona clinic liver cancer; C: comparator; FOLFOX: oxaliplatin, leucovorin, and 5-fluorouracil (IV and continuous IV doses); FOLFOX4: oxaliplatin, leucovorin, and 5-fluorouracil (IV and continuous IV doses); FOLFOX6: oxaliplatin, leucovorin, and 5-fluorouracil (IV and continuous IV doses); GEMOX: gemcitabine and oxaliplatin; HAIC: hepatic arterial infusion chemotherapy; I: intervention; mFOLFOX: modified FOLFOX, levofolinic acid, oxaliplatin, and 5-fluorouracil (continuous IV dose); NS, not stated; PD-1, programmed death-1; PD-L1, programmed death-ligand 1; RALOX: raltitrexed and oxaliplatin; TACE: transarterial chemoembolization; XELOX: oxaliplatin and capecitabine.

**Table 5:** Intervention details.

Reference	Intervention	Dose	Comparator	Dose
Zhang et al. [17]	Apatinib	500 mg, QD	-	-
Qin et al. [18]	Donafenib	200 mg, bid	Sorafenib	400 mg, bid
Wang et al. [19]	Lenvatinib	8 mg if <60 kg or 12 mg if ≥60 kg, QD	-	-
Ye et al. [20]	Sorafenib after surgery	800 mg, QD ^a^	Sorafenib without surgery	-
Ye et al. [21]	Sorafenib	800 mg, QD ^a^	-	-
Qin et al. [22]	Apatinib	750 mg, QD	Placebo	-
Dong et al. [23]	Cabozantinib	-	Cabozantinib + ICI	-
Bi et al. [24]	Donafenib	200 mg, bid	Donafenib	300 mg, bid
Kong et al. [25]	Apatinib	500 mg QD for first 6 patients, then 250 mg QD	-	-
Yau et al. [26]	Sorafenib	400 mg, bid, four-week cycles	-	-
Qin et al. [27]	Camrelizumab	3 mg/kg, Q2W-Q3W		
Ren et al. [28]	Camrelizumab	3 mg/kg, Q2W-Q3W		
Qin et al. [29]	Pembrolizumab	200 mg, Q3W	Placebo	-
Edeline et al. [30]	Tislelizumab	200 mg, Q3W	-	-
Yang et al. [31]	ADI-PEG20	160 IUm^2^, Q1W	ADI-PEG20	320 IUm^2^, Q1W
Zhu et al. [32]	FOLFOX4	85 mg/m^2^, d1 + 200 mg/m^2^, d1, d2 + 400 mg/m^2^ Bolus IV, d1, d2 + 600 mg/m^2^, CIV, d1, d2, Q2W	-	-
Jiang et al. [33]	XELOX	130 mg/m^2^ d1 + 1000 mg/m^2^ bid d1-d14, Q3W	FOLFOX	85 mg/m^2^, d1 + 200 mg/m^2^, d1, d2 + 400 mg/m^2^ + 600 mg/m^2^ d1, d2, Q2W
Zhang et al. [34]	FOLFOX6	100 mg/m^2^ d1 + 200 mg/m^2^ d1 + 400 mg/m^2^ + 240 mg/m^2^, Q2W	-	-
Qin et al. [35]	FOLFOX4	85 mg/m^2^ d1 + 200 mg/m^2^, d1, d2 + 400 mg/m^2^ + 600 mg/m^2^ d1, d2, Q2W	Doxorubicin	500 mg/m^2^, Q3W
Chen et al. [36]	Sintilimab + anlotinib	200 mg, d1 + 12 mg, po, QD, d1-14, Q3W	-	-
Xu et al. [37]	First-line camrelizumab + apatinib	200 mg (for bodyweight ≥50 kg) or 3 mg/kg (for bodyweight <50 kg) Q2W + 250 mg QD	Second-line camrelizumab + apatinib	200 mg (for bodyweight ≥50 kg) or 3 mg/kg (for bodyweight < 50 kg) Q2W + 250 mg QD
Liu et al. [38]	Camrelizumab + sorafenib	200 mg Q2W + 400 mg QD	Sorafenib	400 mg, QD
Lin et al. [39]	Anlotinib + toripalimab	12 mg, po, qd, d1-14, Q3W + 240 mg, d1, Q3W	-	-
Han et al. [40]	Penpulimab + anlotinib	200 mg Q3W + 8 mg QD, 2 weeks on, 1 week off	-	-
Yuan et al. [41]	Camrelizumab + apatinib	200 mg Q3W + 250 mg QD	-	-
Qin et al. [42]	Atezolizumab + bevacizumab	1200 mg + 15 mg/kg, Q3W	Sorafenib	400 mg, bid
Ren et al. [43]	Sintilimab + IBI305	200 mg + Medi15 mg/kg, d1, Q3W	Sorafenib	400 mg, bid
Jia et al. [44]	Sintilimab + IBI305	200 mg Q3W + 15 mg/kg Q3W	-	-
Zhang et al. [45]	Sintilimab + IBI305	200 mg + 7.5 mg/kg, Q3W	Sintilimab + IBI305	200 mg + 15 mg/kg, Q3W
Li et al. [46]	Camrelizumab + FOLFOX4	3 mg/kg Q2W + 85 mg/m^2^, d1 + 200 mg/m^2^, d1, d2 + 400 mg/m^2^ Bolus IV, d1, d2 + 600 mg/m^2^, CIV, d1, d2, Q2W	-	-

^a^ Median, 5-FU: 5-fluorouracil; ADI-PEG 20: Arginine deiminase-polyethylene glycol; bid: twice a day; CIV: continuous intravenous; FOLFOX: oxaliplatin, leucovorin, and 5-fluorouracil (IV and continuous IV doses); FOLFOX4: oxaliplatin, leucovorin, and 5-fluorouracil (IV and continuous IV doses); FOLFOX6: oxaliplatin, leucovorin, and 5-fluorouracil (IV and continuous IV doses); GEMOX: gemcitabine and oxaliplatin; HAIC: hepatic arterial infusion chemotherapy; mFOLFOX: modified FOLFOX, levofolinic acid, oxaliplatin, and 5-fluorouracil (continuous IV dose); PO: per os; Q1W: weekly; Q2W: every 2 weeks; Q3W: every 3 weeks; Q4W: every 4 weeks; QD: once a day; RALOX: raltitrexed and oxaliplatin; TACE: transarterial chemoembolization; XELOX: oxaliplatin and capecitabine.

**Table 6:** Outcome data.

**Tyrosine Kinase Inhibitor**															
**Reference**	**Intervention**	**Line of Treatment**	**ORR, *n* (%) [95% CI]**			**Median PFS, Months [95% CI]**		**Median DOR, Months [95% CI]**		**Median OS, Months [95% CI]**		**TRAEs, *n* (%)**			
												**≥1 Event, Any Grade**		**≥1 Event, ≥Grade 3**	
			**Definition**	**I**	**C**	**I**	**C**	**I**	**C**	**I**	**C**	**I**	**C**	**I**	**C**
Zhang et al. [17]	Apatinib	First-line	RECIST 1.1	6 (37.5)	-	NR	-	-	-	-	-	-	-	-	-
Qin et al. [22]	Apatinib	First-line	RECIST 1.1	(11) [7–15]	(2) [0–5]	4.5 [3.9–4.7]	1.9 [1.9–2.0]	6.5 (5.3-NR]	-	8.7 [7.5–9.8]	6.8 [5.7–9.1]	250 (97)	92 (71)	199 (77)	25 (19)
Kong et al. [25]	Apatinib	Not specified	RECIST 1.1	9 (40.9)	-	-	-	-	-	-	-	^e^	^e^	^e^	^e^
Qin et al. [18]	Donafenib	First-line	RECIST 1.1	15 (4.6)	9 (2.7)	3.7	3.6	-	-	12.1	10.3	314 (94)	321 (97)	125 (38)	165 (50)
Bi et al. [24]	Donafenib	First-line	RECIST 1.1	4 (4.8) ^a^	-	-	-	-	-	-	-	^d^	^d^	^d^	^d^
Wang et al. [19]	Lenvatinib	First- or second-line	RECIST 1.1	12 (22)	-	5.6 [4.3–6.8]	-	-	-	NR	-	AEs: 52 (92.9)	-	AEs: 11 (21.1)	-
Ye et al. [20]	Sorafenib	First-line	RECIST 1.0	-	-	6.8 [4.8–7.6]	5.7 [4.7–7.6]	-	-	11.3	9.9 [8.0–12.2]	36 (32.4)	59 (26.8)	5 (4.5)	7 (3.2)
Ye et al. [21]	Sorafenib	First-line	-	-	-	6.0 ^b^ 6.8 ^c^	-	-	-	10.6 ^b^ 7.9 ^c^	-	167 (50.5)	-	20 (6.0)	-
Yau et al. [26]	Sorafenib	First-line	RECIST	4 (7.8)	-	3 [3–17]	-	-	-	5 [3–17]	-	^f^	^f^	^f^	^f^
Dong et al. [23]	Cabozantinib	Second-line or beyond	-	1 (9.1)	0	-	-	-	-	5.36	12.32	20 (90.9)		12%	
**PD-1/PD-L1 Inhibitor**															
**Reference**	**Intervention**	**Line of Treatment**	**ORR, *n* (%) [95% CI]**			**Median PFS, Months [95% CI]**		**Median DOR, Months [95% CI]**		**Median OS, Months [95% CI]**		**TRAEs, *n* (%)**			
												**≥1 Event, Any Grade**		**≥1 Event, ≥Grade 3**	
			**Definition**	**I**	**C**	**I**	**C**	**I**	**C**	**I**	**C**	**I**	**C**	**I**	**C**
Qin et al. [27]	Camrelizumab	Second-line	RECIST 1.1	32 (14.7) [10.3–20.2]		2.1 [2.0–3.2]	-	NR [3.7–14.0]	-	13.8 [11.5–16.6]	-	-	-	47 (22) ^h^	-
Ren et al. [28]	Camrelizumab	Second-line	RECIST 1.1	-	-	-	-	NR [2.5–30.5 + ] ^i^		14.2 [11.5–16.3]		^i^	^i^	^i^	^i^
Qin et al. [29]	Pembrolizumab	Second-line	RECIST 1.1	(13.7)	(1.3)	2.6 [1.5–2.8]	2.3 [1.4–2.8]	23.9	5.6	14.6 [12.6–18.0]	13.0 [10.5–15.1]	66.9	49.7	14.4	5.9
Edeline et al. [30]	Tislelizumab	Second-line	RECIST 1.1	(13.6) [9.5, 18.7]	-	2.7 [1.6, 2.8]	-	NR	-	13.5 [10.9–15.8]	-	62.6	-	13.6	-
**Chemotherapy**															
**Reference**	**Intervention**	**Line of Treatment**	**ORR, *n* (%) [95% CI]**			**Median PFS, Months [95% CI]**		**Median DOR, Months [95% CI]**		**Median OS, Months [95% CI]**		**TRAEs, *n* (%)**			
												**≥1 Event, Any Grade**		**≥1 Event, ≥Grade 3**	
			**Definition**	**I**	**C**	**I**	**C**	**I**	**C**	**I**	**C**	**I**	**C**	**I**	**C**
Yang et al. [31]	ADI-PEG 20	First- or second-line	RECIST	0	-	1.8 (1.8–3.5)	1.9 (1.8–3.5)	-	-	8.4 [3.6–13.1]	6.2 [2.8–9.6]	-	-	-	-
Zhu et al. [32]	FOLFOX4	Second-line or beyond	RECIST	4 (20)	-	-	-	-	-	5	-	-	-	-	-
Jiang et al. [33]	XELOX	First-line	RECIST	(14.8)	(15.4)	-	-	-	-	3 years: 7.41%	3 years: 3.88%	-	-	-	-
Zhang et al. [34]	FOLFOX6	First- or second-line and beyond	RECIST	(16.1)	-	-	-	-	-	9.7 (4.5-NR)	-	-	-	-	-
Qin et al. [35]	FOLFOX4	First- or second-line	RECIST 1.0	(8.2) [4.6–13.1]	(2.7) [0.9–6.1]	2.9 [2.4–3.5]	1.8 [1.6–2.3]	-	-	6.4 [5.3–7.0]	5.0 [4.2–6.0]	173 (94.5)	159 (91.4)	102 (55.7)	79 (45.4)
**PD-1/PD-L1 Inhibitor + Tyrosine Kinase Inhibitor**															
**Reference**	**Intervention**	**Line of Treatment**	**ORR, *n* (%) [95% CI]**			**Median PFS, Months [95% CI]**		**Median DOR, Months [95% CI]**		**Median OS, Months [95% CI]**		**TRAEs, *n* (%)**			
												**≥1 Event, Any Grade**		**≥1 Event, ≥Grade 3**	
			**Definition**	**I**	**C**	**I**	**C**	**I**	**C**	**I**	**C**	**I**	**C**	**I**	**C**
Chen et al. [36]	Sintilimab + anlotinib	First-line	RECIST 1.1	6 (42.9)	-	NR, 6 months: 78.8% [38.1–94.3]	-	NR	-	NR	-	-	-	6 (37.5)	-
Xu et al. [37]	Camrelizumab + apatinib	First- or second-line	RECIST 1.1	24 (34.3) [23.3–46.6]	27 (22.5) [15.4–31.0]	5.7 [5.4–7.4]	5.5 [3.7–5.6]	14.8 [5.5-NR]	NR	NR, 18-months estimated: 58.1% [45.4–68.9]	NR, 18-months estimated: 56.5% 45.7–66.0]	69 (98.6)	120 (100)	55 (77.4)	92 (76.7)
Liu et al. [38]	Camrelizumab + sorafenib	Not specified	mRECIST	6 (24.0)	2 (4.0)	8.0	6.4	-	-	-	-	AEs: 8 (32)	11 (22)	-	-
Lin et al. [39]	Anlotinib + toripalimab	First-line	mRECIST	(21.4) [4.7–50.8]	-	-	-	-	-	-	-	14 (77.8)	-	7 (38.9)	-
Han et al. [40]	Penpulimab + anlotinib	First-line	RECIST 1.1	9 (31.0)	-	8.8 [4.0–12.3]	-	NE	-	NR	-	28 (90.3)	-	6 (19.4)	-
Yuan et al. [41]	Camrelizumab + apatinib	Second-line	mRECIST	(31.9)	-	6.6	-	-	-	NR: 1 year 62.3%	-	-	-	-	-
**PD-1/PD-L1 Inhibitor + Bevacizumab or Biosimilar**															
**Reference**	**Intervention**	**Line of Treatment**	**ORR, *n* (%) [95% CI]**			**Median PFS, Months [95% CI]**		**Median DOR, Months [95% CI]**		**Median OS, Months [95% CI]**		**TRAEs, *n* (%)**			
												**≥1 Event, Any Grade**		**≥1 Event, ≥Grade 3**	
			**Definition**	**I**	**C**	**I**	**C**	**I**	**C**	**I**	**C**	**I**	**C**	**I**	**C**
Qin et al. [42]	Atezolizumab + bevacizumab	First-line	RECIST 1.1	(24.6) [17.5–32.9]	(6.7) [1.9–16.2]	5.7 [4.2–8.3]	3.2 [2.6–4.8]	-	-	NR [13.5-NR]	11.4 [6.7-NR]	119 (90.2)	54 (93.1)	60 (45.4)	23 (39.6)
Ren et al. [43]	Sintilimab + IBI305	First-line	RECIST 1.1	75 (21) [17–25]	7(4) [2–8]	4.6 [4.1–5.7]	2.8 [2.7–3.2]	NE [NE-NE]	9.8 [2.8–NE]	NR	10.4 [8.5–NR]	376 (98.9) ^j^	181 (97.8) ^j^	209 (55) ^j^	89 (48.1) ^j^
	Sintilimab + IBI305	First-line	RECIST 1.1	(25)	-	8.4 [5.6 –NR]	-	-	-	NR, 6-months: 87.10%	-	18 (75)	-	6 (25)	-
Zhang et al. [45]	Sintilimab + IBI305	Not specified	RECIST 1.1	(24.1) [10.3–43.5]	(33.3) [13.3–59.0]	NR, 6-months: 60.5% [36.1–78.0]	NR, 6-months: 75.8% [47.3–90.2]	-	-	-	-	-	-		6 (12.0)-
**Chemotherapy + PD-1/PD-L1 Inhibitor**															
**Reference**	**Intervention**	**Line of Treatment**	**ORR, *n* (%) [95% CI]**			**Median PFS, Months [95% CI]**		**Median DOR, Months [95% CI]**		**Median OS, Months [95% CI]**		**TRAEs, *n* (%)**			
												**≥1 Event, Any Grade**		**≥1 Event, ≥Grade 3**	
			**Definition**	**I**	**C**	**I**	**C**	**I**	**C**	**I**	**C**	**I**	**C**	**I**	**C**
Li et al. [46]	Camrelizumab + FOLFOX4	First-line	RECIST 1.1	(29.4) [15.1–47.5]	-	7.4 [3.9–9.2]	-	6.9 [3.3–11.5]	-	11.7 [8.2–22.0]	-	100%		85.3%	-

^a^ Combined both doses. ^b^ Patients with Child–Pugh A HCC. ^c^ Patients with Child–Pugh B HCC. ^d^ TRAE incidence not reported, but most common AEs that led to dose discontinuation or reductions were hand–foot skin reaction in 10 (9.4%) patients (2 vs. 8), liver dysfunction in 4 (3.8%) patients (1 vs. 3), and leukopenia in 2 (1.9%) patients (1 vs. 1). ^e^ TRAE incidence not reported, but AEs in the 22 patients mainly consisted of HFSR (81.8%), diarrhea (77.3%), hypertension (63.6%), fatigue (59.1%), hoarseness (54.5%), and nausea (50%). Grade 3 or 4 drug-related AEs mainly included hypertension (27.3%), HFSR (13.6%), and thrombocytopenia (9.1%). ^f^ TRAE incidence not reported, but the following were reported: hematologic toxicities (17% vs. 33%) and grade 3 or 4 nonhematologic toxicities (47% vs. 47%); grade 3 or 4 liver function derangement (56% vs. 73%). ^h^ Grade 3/4 TRAEs were reported for all patients, but TRAEs of any grade were not reported for the total population. ^i^ DOR data are for all patients; total TRAE incidence was not reported but described for specific AE types. ^j^ Data values are for TEAEs. ADI-PEG 20: Arginine deiminase-polyethylene glycol; AE: adverse event; C: comparator; CI: confidence interval; DOR: duration of response; FOLFOX: oxaliplatin, leucovorin, and 5-fluorouracil (IV and continuous IV doses); FOLFOX6: oxaliplatin, leucovorin, and 5-fluorouracil (IV and continuous IV doses); GEMOX: gemcitabine and oxaliplatin; HFSR, hand–foot skin reaction; I: intervention; mFOLFOX: modified FOLFOX levofolinic acid, oxaliplatin, and 5-fluorouracil (continuous IV dose); mRECIST: modified response evaluation criteria in solid tumors; NE: not evaluable; NR: not reached; OS: overall survival; ORR: overall response rate; PD-1, programmed death-1; PD-L1, programmed death-ligand 1; PFS: progression-free survival; RALOX: raltitrexed and oxaliplatin; RECIST: response evaluation criteria in solid tumors; TACE: transarterial chemoembolization; TEAE, treatment-emergent adverse event; TRAE: treatment-related adverse event; XELOX: oxaliplatin and capecitabine.

## Data Availability

All the data, protocol, and other materials used in the review are publicly available and can be found in the information sources stated in the methods.

## Supplementary Materials

The following supporting information can be downloaded at: https://www.mdpi.com/article/10.3390/curroncol29100575/s1, File S1: PRISMA Checklist, File S2: Methods, File S3: PRISMA Checklist for Abstracts.
