# Supplementary material for: First- and Second-Line Treatments for Patients with Advanced Hepatocellular Carcinoma in China: A Systematic Review
Source: Curr Oncol. 2022 Sep 30;29(10):7305–26. doi: 10.3390/curroncol29100575 (PMC9600684; doi:10.3390/curroncol29100575)
Supplement: Supplementary file 1 [file curroncol-29-00575-s001.zip › File S2. Search strategies.pdf]

## File S2. Search strategies

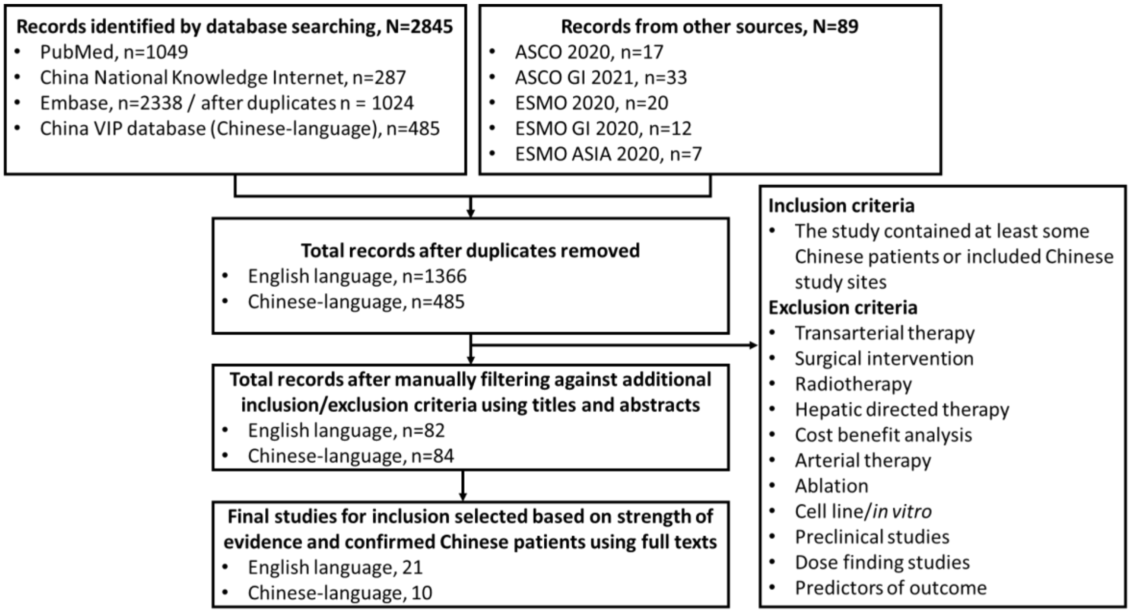


## Search strings

#1 "Carcinoma, Hepatocellular"[Mesh] OR Hepatocellular Carcinoma*[tiab] OR Liver Cell Carcinoma*[tiab] OR Liver Cancer*[tiab] OR Hepatoma*[tiab] OR HCC[tiab] OR Liver Carcinoma*[tiab] 148,426

#2 "Programmed Cell Death 1 Receptor"[Mesh] OR "Programmed Cell Death 1 Ligand 2 Protein"[Mesh] OR "B7-H1 Antigen"[Mesh] OR programmed cell death protein-1[tw] OR programmed cell death-ligand 1[tw] OR "PD-1"[tw] OR "PD-L1"[tw] OR "PD1"[tw] OR "PDL1"[tw] OR cytotoxic T lymphocyte-associated protein-4 inhibitor*[tw] OR CTLA-4 inhibitor*[tw] OR CTLA-4 antibod*[tw] OR CTLA-4 anti-bod*[tw] OR "anti-CTLA-4"[tw] OR CTLA4 inhibitor*[tw] OR CTLA4 antibod*[tw] OR CTLA4 anti-bod*[tw] OR "anti-CTLA4"[tw] OR "CTLA-4 Antigen"[Mesh] OR CD152 Antigen*[tw] OR CTLA 4 Antigen*[tw] OR CTLA4 Antigen*[tw]

#3 "Nivolumab"[Mesh] OR Nivolumab[tw] OR Opdivo[tw] OR "ONO-4538"[tw] OR ONO4538[tw] OR "MDX-1106"[tw] OR MDX1106[tw] OR "BMS-936558"[tw] OR BMS936558[tw] OR "atezolizumab" [Supplementary Concept] OR atezolizumab[tw] OR "anti-PDL1"[tw] OR MPDL3280A[tw] OR "MPDL-3280A"[tw] OR Tecentriq[tw] OR "RG-7446"[tw] OR RG7446[tw] OR "camrelizumab" [Supplementary Concept] OR camrelizumab[tw] OR "SHR-1210"[tw] OR SHR1210[tw] OR "sintilimab" [Supplementary Concept] OR sintilimab[tw] OR "IBI 308"[tw] OR IBI308[tw] OR "pembrolizumab" [Supplementary Concept] OR pembrolizumab[tw] OR "SCH-900475"[tw] OR

Keytruda[tw] OR "MK-3475"[tw] OR lambrolizumab[tw] OR mk3475[tw] OR sch900475[tw] OR tislelizumab[tw] OR "bgb a317"[tw] OR bgba317[tw] OR "toripalimab" [Supplementary Concept] OR toripalimab[tw] OR "js 001"[tw] OR js001[tw] OR "tab 001"[tw] OR tab001[tw] OR "cemiplimab" [Supplementary Concept] OR cemiplimab[tw] OR REGN2810[tw] OR "REGN 2810"[tw] OR "cemiplimab rwlc"[tw] OR libtayo[tw] OR "sar 439684"[tw] OR sar439684[tw] OR "avelumab" [Supplementary Concept] OR avelumab[tw] OR "MSB-0010682"[tw] OR MSB0010682[tw] OR bavencio[tw] OR MSB0010718C[tw] OR "MSB-0010718C"[tw] OR MSB10718C[tw] OR "MSB-10718C"[tw] OR "MSB-10682"[tw] OR MSB10682[tw] OR "pf 6834635"[tw] OR pf6834635[tw] OR "pf 06834635"[tw] OR pf06834635[tw] OR "durvalumab" [Supplementary Concept] OR durvalumab[tw] OR "MEDI-4736"[tw] OR MEDI4736[tw] OR Imfinzi[tw] OR Envafolimab[tw] OR "asc 22"[tw] OR asc22[tw] OR "kn 035"[tw] OR kn035[tw] OR Sugemalimab[tw] OR "cs 1001"[tw] OR cs1001[tw] OR "wbp 315"[tw] OR wbp315[tw] OR "wbp 3155"[tw] OR wbp3155[tw] OR GB226[tw] OR "GLS-010"[tw]

#4 "Ipilimumab"[Mesh] OR Ipilimumab[tw] OR Yervoy[tw] OR "MDX 010"[tw] OR MDX010[tw] OR "MDX-CTLA-4"[tw] OR "tremelimumab" [Supplementary Concept] OR tremelimumab[tw] OR ticilimumab[tw] OR "CP 675"[tw] OR CP675[tw] OR "CP-675206"[tw] OR CP675206[tw] OR "Cytokine-Induced Killer Cells"[Mesh] OR "CIK Cell*"[tw] OR Cytokine-Induced Killer Cell*[tw] OR Lymphocyte Activated KillerCell*[tw] OR Stimuvax[tw] OR "L-BLP25"[tw] OR "Belagenpumatucel-L"[tw] OR "belagenpumatucel L" [Supplementary Concept] OR Lucanix[tw] OR AK105[tw]

#5 "Sorafenib"[Mesh] OR "Erlotinib Hydrochloride"[Mesh] OR "cabozantinib" [Supplementary Concept] OR "tepotinib" [Supplementary Concept] OR "regorafenib" [Supplementary Concept] OR "ARQ 197" [Supplementary Concept] OR Tyrosine kinase inhibitor*[tw] OR Sorafenib[tw] OR Nexavar[tw] OR "BAY 43-9006"[tw] OR "BAY 439006"[tw] OR tivantinib[tw]

#6 "Drug Therapy"[Mesh] OR "drug therapy" [Subheading] OR "Antineoplastic Agents"[Mesh:NoExp] OR "Antibiotics, Antineoplastic"[Mesh] OR "Anticarcinogenic Agents"[Mesh] OR "Antimetabolites, Antineoplastic"[Mesh] OR "Antimitotic Agents"[Mesh] OR "Antineoplastic Agents, Alkylating"[Mesh] OR "Antineoplastic Agents, Hormonal"[Mesh] OR "Antineoplastic Agents, Phytogenic"[Mesh] OR "Myeloablative Agonists"[Mesh] OR "Poly(ADP-ribose) Polymerase Inhibitors"[Mesh] OR "Topoisomerase Inhibitors"[Mesh] OR "Antibiotics, Antineoplastic" [Pharmacological Action] OR "Antimetabolites, Antineoplastic" [Pharmacological Action] OR "Antineoplastic Agents" [Pharmacological Action] OR "Antineoplastic Agents, Alkylating" [Pharmacological Action] OR "Antineoplastic Agents, Hormonal" [Pharmacological

Action] OR "Antineoplastic Agents, Phytogenic" [Pharmacological Action] OR "Cyclophosphamide"[Mesh] OR "Daunorubicin"[Mesh] OR "Doxorubicin"[Mesh] OR "Mitoxantrone"[Mesh] OR "Methotrexate"[Mesh] OR "Vincristine"[Mesh] OR "Vindesine"[Mesh] OR "Etoposide"[Mesh] OR "Teniposide"[Mesh] OR "Dexamethasone"[Mesh] OR "Cisplatin"[Mesh] OR "Carboplatin"[Mesh] OR "Oxaliplatin"[Mesh] OR "Paclitaxel"[Mesh] OR "Docetaxel"[Mesh] OR "gemcitabine" [Supplementary Concept]

#7 Cyclophosphamide[tw] OR Sendoxan[tw] OR "B-518"[tw] OR B518[tw] OR Cytophosphan*[tw] OR Cytoxan[tw] OR Endoxan[tw] OR Neosar[tw] OR "NSC 26271"[tw] OR NSC26271[tw] OR Procytox[tw] OR Cyclophosphane[tw] OR Daunorubicin[tw] OR Daunomycin[tw] OR Rubomycin[tw] OR "Dauno Rubidomycine"[tw] OR Rubidomycin[tw] OR Daunoblastin[tw] OR Daunoblastine[tw] OR NSC 82151[tw] OR NSC82151[tw] OR Cerubidine[tw] OR Doxorubicin*[tw] OR Farmiblastina[tw] OR Ribodoxo[tw] OR Rubex[tw] OR Adriamycin[tw] OR Adriblastin[tw] OR Adriblastin*[tw] OR Adriablastin*[tw] OR Adrimedac[tw] OR "DOXO cell"[tw] OR Doxolem[tw] OR Doxotec[tw] OR Myocet[tw] OR Onkodox[tw] OR Mitoxantrone[tw] OR mitoxanthraquinone[tw] OR Mitozantrone[tw] OR DHAQ[tw] OR "NSC 279836"[tw] OR NSC279836[tw] OR "NSC 287836"[tw] OR NSC287836[tw] OR "NSC 299195"[tw] OR NSC299195[tw] OR "NSC 301739"[tw] OR NSC301739[tw] OR "NSC 301739D"[tw] OR NSC301739D[tw] OR Mitroxone[tw] OR Pralifan[tw] OR "CL 232325"[tw] OR CL232325[tw] OR Novantron*[tw] OR Ralenova[tw] OR Onkotrone[tw] OR Methotrexate[tw] OR Amethopterin[tw] OR Mexate[tw] OR Vincristin*[tw] OR Leurocristine[tw] OR cellcristin[tw] OR Citomid[tw] OR Oncovin*[tw] OR Onkocristin[tw] OR Vincasar[tw] OR Farmistin[tw] OR Vintec[tw] OR Vincrisul[tw] OR Vindesin*[tw] OR "Desacetylvinblastine Amide"[tw] OR Eldisine[tw] OR Enison[tw] OR "NSC 245467"[tw] OR NSC245467[tw] OR "Compound 112531"[tw] OR Etoposid*[tw] OR Eposide[tw] OR "Demethyl Epipodophyllotoxin Ethylidine Glucoside"[tw] OR "Eto GRY"[tw] OR Exitop[tw] OR Lastet[tw] OR "NSC 141540"[tw] OR NSC141540[tw] OR Onkoposid[tw] OR Riboposid[tw] OR Toposar[tw] OR Vepesid[tw] OR "VP 16 213"[tw] OR "VP 16213"[tw] OR "VP 16"[tw] OR VP16[tw] OR "Vépéside Sandoz"[tw] OR Celltop[tw] OR Etopos[tw] OR Etomedac[tw] OR Eposin[tw] OR Teniposide[tw] OR "Demethyl Epipodophyllotoxin Thenylidine Glucoside"[tw] OR Vumon[tw] OR "VM 26"[tw] OR VM26[tw] OR "NSC 122819"[tw] OR NSC122819[tw] OR Dexamethasone[tw] OR Methylfluorprednisolone[tw] OR Hexadecadrol[tw] OR Decameth[tw] OR Decaspray[tw] OR Dexasone[tw] OR Dexpak[tw] OR Maxidex[tw] OR Millicorten[tw] OR Oradexon[tw] OR Decaject[tw] OR Hexadrol[tw] OR Cisplatin[tw] OR "cis-Diamminedichloroplatinum"[tw] OR "Platinum Diamminodichloride"[tw] OR "cis Platinum"[tw] OR Dichlorodiammineplatinum[tw] OR "NSC-119875"[tw] OR Platino*[tw]

OR Biocisplatinum[tw] OR Platidiam[tw] OR Carboplatin[tw] OR CBDCA[tw] OR Paraplatin*[tw] OR "cis-Diammine(cyclobutanedicarboxylato)platinum II"[tw] OR Platinwas[tw] OR Ribocarbo[tw] OR Carboplat[tw] OR Neocarbo[tw] OR Carbosin[tw] OR Carbotec[tw] OR Ercar[tw] OR "JM 8"[tw] OR JM8[tw] OR Nealorin[tw] OR "NSC 241240"[tw] OR NSC241240[tw] OR Blastocarb[tw] OR Oxaliplatin*[tw] OR "Oxalato-(1,2-cyclohexanediamine)platinum II"[tw] OR "L-OHP Cpd"[tw] OR "Platinum(II)-1,2-cyclohexanediamine Oxalate"[tw] OR "1,2-Diaminocyclohexane Platinum Oxalate"[tw] OR Eloxatin*[tw] OR "ACT 078"[tw] OR ACT078[tw] OR Paclitaxel[tw] OR Anzatax[tw] OR "NSC 125973"[tw] OR NSC125973[tw] OR Taxol[tw] OR Paxene[tw] OR Praxel[tw] OR Onxol[tw] OR Docetaxel[tw] OR Docetaxol[tw] OR "Taxoltere Metro"[tw] OR "RP 56976"[tw] OR RP56976[tw] OR Taxotere[tw] OR "NSC 628503"[tw] OR gemcitabine[tw] OR dFdCyd[tw] OR difluorodeoxycytidine[tw] OR "2'-deoxy-2'-difluorocytidine"[tw] OR "2',2'-DFDC"[tw] OR "2',2'-difluoro-2'-deoxycytidine"[tw] OR "LY 188011"[tw] OR Gemzar[tw] OR "2'-deoxy-2',2''-difluorocytidine-5'-O-monophosphate"[tw] OR FOLFOX[tw] OR mFOLFOX[tw]

#8 #2 OR #3 OR #4 OR #5 OR #6 OR #7

#9 "China"[Mesh] OR China OR Chinese OR Taiwan OR Hong kong OR Hongkong OR Macau OR Macao OR Beijing OR Shanghai OR Tianjin OR Chongqing OR Inner Mongolia OR Tibet OR Guangxi OR Sinkiang OR Ningxia OR Xinjiang OR Hebei OR Shanxi OR Liaoning OR Jilin OR Heilongjiang OR Jiangsu OR Zhejiang OR Anhui OR Fujian OR Jiangxi OR Shandong OR Henan OR Hubei OR Hunan OR Guangdong OR Hainan OR Sichuan OR Guizhou OR Yunnan OR Shaanxi OR Gansu OR Qinghai

#10 #1 AND #8 AND #9 8628 P+I, China

#11 "Radiotherapy"[Mesh] OR Radiotherap*[tw] OR Radiation[tw] OR TACE[tw] OR "Transhepatic Arterial Chemotherapy and Embolization"[tw] OR Resection[tw]

#12 #10 AND #11 997 P+I+C, China

#13 ("controlled clinical trial"[Publication Type] OR "Controlled Clinical Trials as Topic"[MeSH] OR "Random Allocation"[MeSH] OR "Double-Blind Method"[MeSH] OR "single-blind method"[MeSH] OR "Control Groups"[MeSH] OR "cross-over studies"[MeSH] OR random*[tiab] OR placebo[tiab] OR trial[tiab] OR groups[tiab] OR crossover[tiab] OR cross-over[tiab]) NOT ("Animals"[Mesh] NOT ("Humans"[Mesh] AND "Animals"[Mesh]))

#14 #12 AND #13 381, RCT

#15 "Observational Study" [Publication Type] OR "Observational Studies as Topic"[Mesh] OR "Cohort Studies"[Mesh] OR "Case-Control Studies"[Mesh] OR "Cross-Sectional Studies"[Mesh] OR Cohort[tiab] OR Follow-Up[tiab] OR Longitudinal*[tiab] OR Prospectiv*[tiab] OR Retrospectiv*[tiab] OR Case-Control[tiab] OR Cross-Sectional[tiab] OR case series*[tiab]

#16 #12 AND #15 450, observational studies

#17 #14 OR #16 604, comparative studies
